# Effects of Aleurone Supplementation on Glucose-Insulin Metabolism and Gut Microbiome in Untrained Healthy Horses

**DOI:** 10.3389/fvets.2021.642809

**Published:** 2021-04-12

**Authors:** Berit Boshuizen, Carmen Vidal Moreno de Vega, Lorie De Maré, Constance de Meeûs, Jean Eduardo de Oliveira, Guilherme Hosotani, Yannick Gansemans, Dieter Deforce, Filip Van Nieuwerburgh, Catherine Delesalle

**Affiliations:** ^1^Research Group of Comparative Physiology, Department of Virology, Parasitology and Immunology, Faculty of Veterinary Medicine, Ghent University, Merelbeke, Belgium; ^2^Wolvega Equine Hospital, Oldeholtpade, Netherlands; ^3^Department of Small Animals and Horses, Faculty of Veterinary Medicine, University of Liège, Liège, Belgium; ^4^Cargill R&D Centre Europe, Vilvoorde, Belgium; ^5^Laboratory of Pharmaceutical Biotechnology, Faculty of Pharmaceutical Sciences, Ghent University, Ghent, Belgium

**Keywords:** prebiotic, equine, insulin sensitivity, ferulic acid, betaine, gut health, Firmicutes, Bacteroidetes

## Abstract

Aleurone, a layer of the bran fraction, is deemed to be responsible for the positive health effects associated with the consumption of whole-grain products. Studies on rodents, pigs, and humans report beneficial effects of aleurone in five main areas: the reduction of oxidative stress, immunomodulatory effects, modulation of energy management, digestive health, and the storage of vitamins and minerals. Our study is the first aleurone supplementation study performed in horses. The aim of this study was to investigate the effect of an increase in the dose levels of aleurone on the postprandial glucose-insulin metabolism and the gut microbiome in untrained healthy horses. Seven adult Standardbred horses were supplemented with four different dose levels of aleurone (50, 100, 200, and 400 g/day for 1 week) by using a Latin square model with a 1-week wash out in between doses. On day 7 of each supplementation week, postprandial blood glucose-insulin was measured and fecal samples were collected. 16S ribosomal RNA (rRNA) gene sequencing was performed and QIIME2 software was used for microbiome analysis. Microbial community function was assessed by using the predictive metagenome analysis tool Phylogenetic Investigation of Communities by Reconstruction of Unobserved States (PICRUSt) and using the Metacyc database of metabolic pathways. The relative abundancies of a pathway were analyzed by using analysis of composition of microbiomes (ANCOM) in R. There was a significant dose-dependent increase in the postprandial time to peak of glucose (*p* = 0.030), a significant delay in the time to peak of insulin (*p* = 0.025), and a significant decrease in both the insulin peak level (*p* = 0.049) and insulin area under the curve (AUC) (*p* = 0.019) with increasing dose levels of aleurone, with a consideration of 200 g being the lowest significant dose. Alpha diversity and beta diversity of the fecal microbiome showed no significant changes. Aleurone significantly decreased the relative abundance of the genera Roseburia, Shuttleworthia, Anaerostipes, Faecalibacter, and Succinovibrionaceae. The most pronounced changes in the relative abundance at phyla level were seen in Firmicutes and Verrucomicrobia (downregulation) and Bacteroidetes and Spirochaetes (upregulation). The PICRUSt analysis shows that aleurone induces a downregulation of the degradation of L-glutamate and taurine and an upregulation of the three consecutive pathways of the phospholipid membrane synthesis of the Archaea domain. The results of this study suggest a multimodal effect of aleurone on glucose-insulin metabolism, which is most likely to be caused by its effect on feed texture and subsequent digestive processing; and a synergistic effect of individual aleurone components on the glucose-insulin metabolism and microbiome composition and function.

## Introduction

Whole-grain products have been incorporated in human and animal diets for some decades, which not only meet the conventional nutritional needs such as dietary fiber content but also allow for an improvement of gut microbiome, a stimulation of increased metabolic health, lower risk of cardiovascular disease mortality, and a lower risk of cancer (1–8).

Aleurone is known as the layer within the bran fraction of wheat, rye and oat, that is deemed to be responsible for positive health effects associated with the consumption of whole-grain products (9–11). Aleurone comprises a single-cell layer located between the endosperm and the seed coat of the wheat kernel (12, 13). The predominant function inside the wheat kernel is to regulate the exchange of nutrients that are stored inside the endosperm to allow for the seed embryo germination (14, 15).

When conventional grain milling techniques are applied, the aleurone fraction remains attached to the bran fraction (16), which leads to a low oral bioavailability (17–19). Recently, however, advanced milling and dry-fractionation techniques have been developed to separate the aleurone from the bran fraction [(20–22); Bühler Group, Switzerland]. This allows for a selective incorporation of pure aleurone in both food and feed (23). Based on the applied fractionation technique, aleurone is available in different purity forms: Aleurone Standard Preparation 1 (ASP-01) or a more purified form ASP-02 [(24); Table 1].

**Table 1:** Overview of the composition of aleurone standard preparation 1 (ASP-01) in grams or milligrams per 100 g dry matter (DM) [adapted from Buri et al. (24)].

**Component**	**ASP-01**
*Crude protein (N × 5.70)*	16.9 g/100 g DM^a^
*Crude fat*	5.8 g/100 g DM
Polyunsaturated fatty acids	66% of crude fat
Monounsaturated fatty acids	18% of crude fat
Saturated fatty acids	16% of crude fat
*Total dietary fiber*	54.1 g/100 g DM
Water-insoluble dietary fiber	50.0 g/100 g DM
Water-soluble dietary fiber	4.1 g/100 g DM
*Crude ash*	9.3 g/100 g DM
Phosphorous	1.9 g/100 g DM
Potassium	1.9 g/100 g DM
Magnesium	0.8 g/100 g DM
Calcium	76.2 mg/100 g DM
Iron	21.3 mg/100 g DM
Zinc	11.4 mg/100 g DM
Sodium	1.7 mg/100 g DM
*Vitamins*	>29 mg/100 g DM
B_1_ (thiamine)	1.6 mg/100 g DM^b^
B_2_ (riboflavin)	0.3 mg/100 g DM
B_3_ (niacin)	24 mg/100 g DM
B_6_ (pyridoxine)	0.3 mg/100 g DM
B_9_ (folate)	0.8 mg/100 g DM
E (α-tocopherol)	2.0 mg/100 g DM
*Phytic acid (4,5,6-IP)*	6.9 g/100 g DM

a *DM, dry matter*.

b *Values correspond to Lopez et al. (51)*.

Aleurone is a biomatrix that contains several different bioactive phytochemicals, such as antioxidants [e.g., ferulic acid (FA)], osmolytes (e.g., betaine), vitamins (e.g., thiamin), essential amino acids (e.g., lysine), and minerals (24, 25). These key components are embedded in an arabinoxylan matrix (26, 27), which represents the fiber fraction of aleurone. The most represented polysaccharides in the fiber fraction are arabinoxylan (65%) and β-glucans (29%) while cellulose plays a minor role (25, 28). The dietary fibers that are present in the aleurone layer are mostly insoluble (26, 29, 30). The arabinose residues of arabinoxylan are generally highly substituted with phenolic compounds, which are either simple phenolic acids, such as FA, p-coumaric acid, sinapic acid, syringic acid, vanillic acid, alkylresorcinols, or complex phenols, such as lignin and lignans (27). FA is deemed to be the most important phenolic compound and is well-known both as a potent antioxidant (31, 32) and as a molecule that can modulate the insulin sensitivity in obese individuals by increasing the expression of the insulin receptor substrate-1 (IRS-1), phosphatidylinositol 3-kinase (PI3K), and protein kinase B (Akt) (33). The total antioxidant capacity of different wheat fractions is highly associated with the FA content (34). FA is approved in some countries for the treatment of cardiovascular and cerebrovascular diseases (35, 36). Due to its predominantly bound form to the arabinoxylan layer, FA can be viewed as a structural component of the cell wall. This probably explains the relatively poor bioavailability of FA from the standard wheat bran when compared to aleurone (17, 37, 38). Additionally, after oral uptake, the bioavailability of FA, like the other aleurone components, depends on the efficiency with which it is released from the fiber food matrix when passing through the GI tract (19). Before the absorption of components into the circulation can take place, they need to be released from the aleurone tissue matrix by either intestinal mucosal enzymes or the intestinal microbiome (39–41).

Another important aleurone component is betaine, which is also known as trimethylglycine (42). Betaine has been studied across many species mainly focusing on its effects on energy metabolism as an endpoint. In both humans and pigs, it has been shown that the feeding of aleurone-enriched diets significantly increases plasma betaine levels (23, 43, 44). Also, when aleurone was incorporated in a bread product, postprandial betaine plasma levels significantly rose (11). Blood plasma betaine steady states are achieved within a few days of dietary intake (45). Betaine is a neutral, zwitterionic compound (43) and a methyl derivative of glycine (45). Several studies across animal species and humans showed that betaine had an influence on the glucose and lipid metabolism and had anti-inflammatory properties (46–48). Betaine insufficiency is associated with lipid metabolism disorders, diabetes, and metabolic syndrome (43, 49). In a study of inducing metabolic syndrome in rats by employing a high-fructose diet, betaine was shown to reduce systemic inflammation, the insulin resistance (IR), and the lipid accumulation (50). This effect was partly attributed to an anti-inflammatory effect of betaine, which improves insulin signaling. Betaine is also reported to act as a lipotropic agent, which prevents or reduces the fat accumulation in the liver by enhancing hepatic lipid export and fatty acid oxidation in high-fat diet-fed rats, and the supplementation has been shown to decrease the low-density lipoprotein (LDL) cholesterol levels in humans (44, 48, 52). Though other compounds within the aleurone fraction could also be responsible for this effect (53), an evidence accumulates that betaine has an important role (48, 50). Animal studies focusing on betaine supplementation report enhanced carcass characteristics and anabolic endocrine profiles such as growth hormone (GH), insulin-like growth factor 1 (IGF-1), and insulin (54–56). It is suggested that betaine stimulates the GH secretion and insulin and IGF-1 receptor signaling (57) by modulating homocysteine thiolactone (HT) pathways.

Studies performed in the different animal species report the beneficial effects of aleurone in the five main areas: the reduction of oxidative stress (34, 58, 59), immunomodulatory effects (9), energy management (23, 44, 57), digestive health (60–62), and the storage of vitamins and minerals (24, 63). To date, no aleurone studies are available for horses. As mentioned in a previous study, the bioavailability of the key components of the aleurone fraction depends on how good the organism succeeds in releasing them from the arabinoxylan containing a fiber food matrix (19). This is achieved by either intestinal mucosal enzymes or intestinal bacterial enzymes (39, 40). In the gut itself, a microbial attack of covalently bound phenolic acids in the aleurone fraction is expected to enhance their release (64). It is not known whether animal species, such as horses, with a profound hindgut fermentative capacity can accomplish a more efficient absorption of the aleurone components. Interestingly, there is also a demonstrated appreciable microbial activity in the equine small intestine (65–67).

The main goal of this current study is to report the effect of increasing doses of aleurone supplementation in healthy untrained horses, focusing on two endpoints: the postprandial glucose and insulin response and shifts in the gut microbiome composition. We hypothesize that aleurone supplementation to non-trained horses dose dependently modulates the postprandial glucose and insulin dynamics and induces shifts in the gut microbiome.

## Materials and Methods

### Animals and Study Design

Seven healthy untrained Standardbred horses (age 4–7 years, 4 ♀, 3♂) were housed in individual boxes (14 m^2^) on wood shavings and with *ad libitum* access to tap water and good quality hay. The amount of hay consumed by each horse was recorded daily. The composition of the hay batch was analyzed employing a Weende analysis. The horses were turned out in sand paddocks 2 h a day. Four different wheat aleurone ASP-01 dose levels were tested (50, 100, 200, and 400 g/day) for 10 consecutive weeks. Throughout this study, we will refer to the wheat aleurone ASP-01 as simply “aleurone.” Aleurone was supplemented at different dose levels following a Latin square design (Table 2). Each supplemented dose was fed for 7 consecutive days followed by a 1-week wash out during which no aleurone was supplemented. Two batches of concentrate feed were manufactured: one pelletized blanco batch in which aleurone was replaced by wheat bran and a pelletized batch containing 20% aleurone (Supplementary Table 1). Both the batches were mixed to achieve the proper aleurone dose. Horses were fed a concentrate meal twice a day at 8 a.m. and 8 p.m. The complete aleurone dose was provided at 8 a.m. Horses were allowed to adapt to the blanco concentrate feed for 4 weeks prior to the start of the study. Horses were checked for their vital signs, such as heart rate, respiratory rate, rectal temperature, the color of mucous membranes, capillary refill time, appetite, and the consistency of stools twice a day. The study was approved by the Animal Ethics Committee of the Ghent University EC 2014.14.

**Table 2:** Latin square design of the experiment.

	**W1**	**W2**	**W3**	**W4**	**W5**	**W6**	**W7**	**W8**	**W9**	**W10**
H1	0	50	0	100	0	200	0	400	0	0
H2	0	100	0	200	0	400	0	50	0	0
H3	0	200	0	400	0	50	0	100	0	0
H4	0	400	0	50	0	100	0	200	0	0
H5	0	0	50	0	100	0	200	0	400	0
H6	0	0	100	0	200	0	400	0	50	0
H7	0	0	400	0	50	0	100	0	200	0

*H1–H7 represent the horses; W, week; H, horse. Doses of aleurone in grams*.

### Postprandial Glucose and Insulin Measurements

On day 7 of each supplementation week, horses were subjected to a postprandial glucose and insulin follow-up. A catheter was inserted into the jugular vein (over the needle, 16G, MILA International, KY, USA), and blood was sampled when feeding started (T0) and every 30 min for 4 consecutive hours and after that every 60 min for another 4 consecutive hours. Blood was collected in NaF-coated tubes for an immediate glucose level assessment (Alphatrak^®^, Zoetis, Belgium). Regarding insulin, blood was collected in heparin-coated tubes at T0 and every 10 min (1 h) followed by every 30 min (4 h). Insulin levels were assessed on the same day by performing a chemiluminescent immunoassay (CIA) [IMMULITE^®^ 1000 Immunoassay System (Siemens Healthcare Diagnostics Inc., Tarrytown, NY, USA)]. On day 7 of the week in which 400 g aleurone was supplemented, blood was taken from the jugular vein (Vacutainer system) for the performance of a routine blood examination including the complete blood count (CBC) and clinical biochemistry.

### Metagenomics

On day 7 of each week, the fecal samples were collected from the rectum. The samples were immediately frozen in dry ice and stored at −80°C until they were processed. The samples collected at the beginning of the trial, collected on the last day of the trial (7 days after the last aleurone supplementation for all horses), collected at day 7 of a week in which aleurone was supplemented, and collected at day 7 of a week in which no aleurone was supplemented are labeled as “blanco horses,” “posttrial horses,” “aleurone supplemented horses,” and “non-supplemented horses,” respectively.

The extraction of DNA was performed by using the QIAamp Fast DNA Stool Mini Kit (QIAGEN, Hilden, Germany). About 10 g of each fecal sample were homogenized in a 90-ml phosphate buffered saline (PBS) by using a stomacher and filtered through a cell strainer (70 μm). After centrifugation, fecal pellets were washed twice with PBS before commencing the extraction of DNA by using the manufacturer's recommendations.

The quantity and quality of DNA were assessed by using a spectrophotometer [NanoDrop 1000 (Thermo Scientific, Wilmington, DE, USA)]. The metagenomics analysis was performed via 16S rRNA amplicon sequencing. One primer pair for the V3 and V4 region of the 16S rRNA gene was used for 25 PCR cycles to create a single amplicon of approximately 460 bp. These primers are described by Klindworth et al. (68) as the most promising bacterial primer pair for 16S sequencing. The gene-specific primers were appended with the adapter sequences that are compatible with a subsequent index PCR that attaches dual indices and Illumina sequencing adapters by using the Illumina Nextera XT Index Kit. By this way, up to 96 libraries can be created and pooled together for sequencing. Libraries are quantified by using quantitative PCR (qPCR) with primers on the required Illumina library adaptor sequences (following the Illumina qPCR quantification protocol guide) and pooled in an equimolar manner. The equimolar pool is denatured and diluted by following Illumina protocols to produce a final 4.5 pM sequencing library. About 20% denatured Illumina PhiX Control V3 library was admixed to increase the sequence diversity of this final library. Cluster generation and 2 x 300 paired-end sequencing is performed in one Illumina Miseq flow cell. Using the Illumina Miseq 300-bp paired-end sequencing, paired-end reads with overlapped ends are generated. The overlapping reads can be stitched to form high quality, full length reads of the V3 and V4 region.

### Glucose and Insulin Data Analysis

All data were subject to an outlier analysis. Values more than 1.5 times the interquartile range were identified as outliers and the identified values were removed from the analysis (69). For responses where data was collected over the aleurone dosage levels of 50–400 g/day, statistical modeling of data was performed by using a mixed-model analysis using the PROC MIXED procedure in SAS (Version 9.3, SAS Institute Inc., Cary, NC, USA). The study design was an extended Latin square according to the model:

$$\begin{array}{l} {\text{Y}}_{\text{ijkl}}=\mu +\text{covariate}+{\text{S}}_{\text{i}}+{\text{H}\left(\text{S}\right)}_{\text{ij}}+{\text{W}\left(\text{S}\right)}_{\text{ik}}+\tau_{\text{l}}+\;\varepsilon_{\text{ijkl}}, \end{array}$$

where, Y_ijkl_ = the specific trait measured for each experimental unit, μ = overall mean for the specific trait, covariate = covariate value for each specific response after the first week on control diet, S_i_ = random square effect (i = I or II), H(S)_ij_ = random horse within square effect (j = I, II, III, …, VIII), W(S)_ik_ = random week within square effect (k = I, II, III, IV), τ_l_ = fixed effect of treatment (l = dose I, dose II, dose III, and dose IV), and ε_ijkl_ = residual error.

For repeated measurements of blood glucose and insulin, the fixed effect of time was added to the abovementioned model as well as the interaction effect of treatment by time and horse was identified as the subject for the random covariance of time effect. Preplanned contrasts were used to estimate regression coefficients over the aleurone dose level and to determine significant relationships for (1) a linear effect, (2) quadratic effect, and (3) cubic effect of aleurone supplementation. No significant effects were found for cubic and quadratic effects; therefore, only linear effects are reported.

### Microbiome Data Analysis

Data preparation and metagenomics analyses both were done by using QIIME2 (v2020.2) (70, 71) unless otherwise mentioned. This includes sequenced read-pair quality trimming, mergence into reconstructed amplicons, operational taxonomic unit (OTU) picking, taxonomic assignment, and phylogenetic reconstruction. To build OTU tables and trees, open-reference OTU picking was performed against the Greengenes 16S reference collection (release 13.8) (72). With the exception of sample 52 (collected in a non-supplemented week of horse number 6), the 16S fragment sequencing depth across all the samples ranged from 28,619 to 128,871. Due to insufficient quality, sample 52 was removed from the analyses. Each downstream diversity metric requiring normalization by a rarefaction was calculated by using a sampling depth ranging between 16,253 and 34,211, corresponding to the lowest sequencing depth of the samples in each comparison we investigated. A rarefaction plot (Supplementary Figure 1) shows the observed OTU saturation after random sampling up to 50,000 sequenced features in each individual sample. To verify if the sequencing was adequate and reached feature saturation, we visually inspected rarefaction plots depicting the number of uniquely identified features at increasing sequencing depth for all individual samples. Alpha diversity metrics, such as Shannon's Diversity, Faith's Phylogenetic Diversity, and the observed OTUs were calculated for each comparison of sample groups and statistically analyzed by using the Kruskal–Wallis test. Beta diversity metrics, such as Weighted UniFrac, Unweighted UniFrac, and Bray–Curtis, were also calculated for each comparison of sample groups and statistically analyzed with a PERMANOVA test to assess the significance of the differences. To identify the taxonomic features that were differentially abundant between the conditions, an analysis of the composition of the microbiome was done in R by using analysis of composition of microbiomes (ANCOM) (v2.1) (73), including the horse identity as a blocking factor in the design. According to the recommendations of the software's authors, the minimum threshold for significantly different taxa was set at 70% for the W statistic (W_0.7_). Fold change (FC) was calculated by using the mean centered log ratio difference. Additionally, the microbial community function was assessed by using the predictive metagenome analysis provided by PICRUSt (74) using the Metacyc (75) metabolic pathways database. To identify differential pathways between the conditions, the resulting pathway abundancies were analyzed by using ANCOM in R.

## Results

Vital signs were monitored on a daily basis throughout the entire trial; respiratory rate, heart rate, temperature, and capillary refill time were always within the reference range. The color of mucous membranes, appetite, and the consistency of stool were considered normal throughout this study.

### CBC and Clinical Biochemistry

No parameters were out of the reference range for both CBC and clinical biochemistry for none of the horses that received 400 g of aleurone.

### Glucose and Insulin

**Figure 1:**
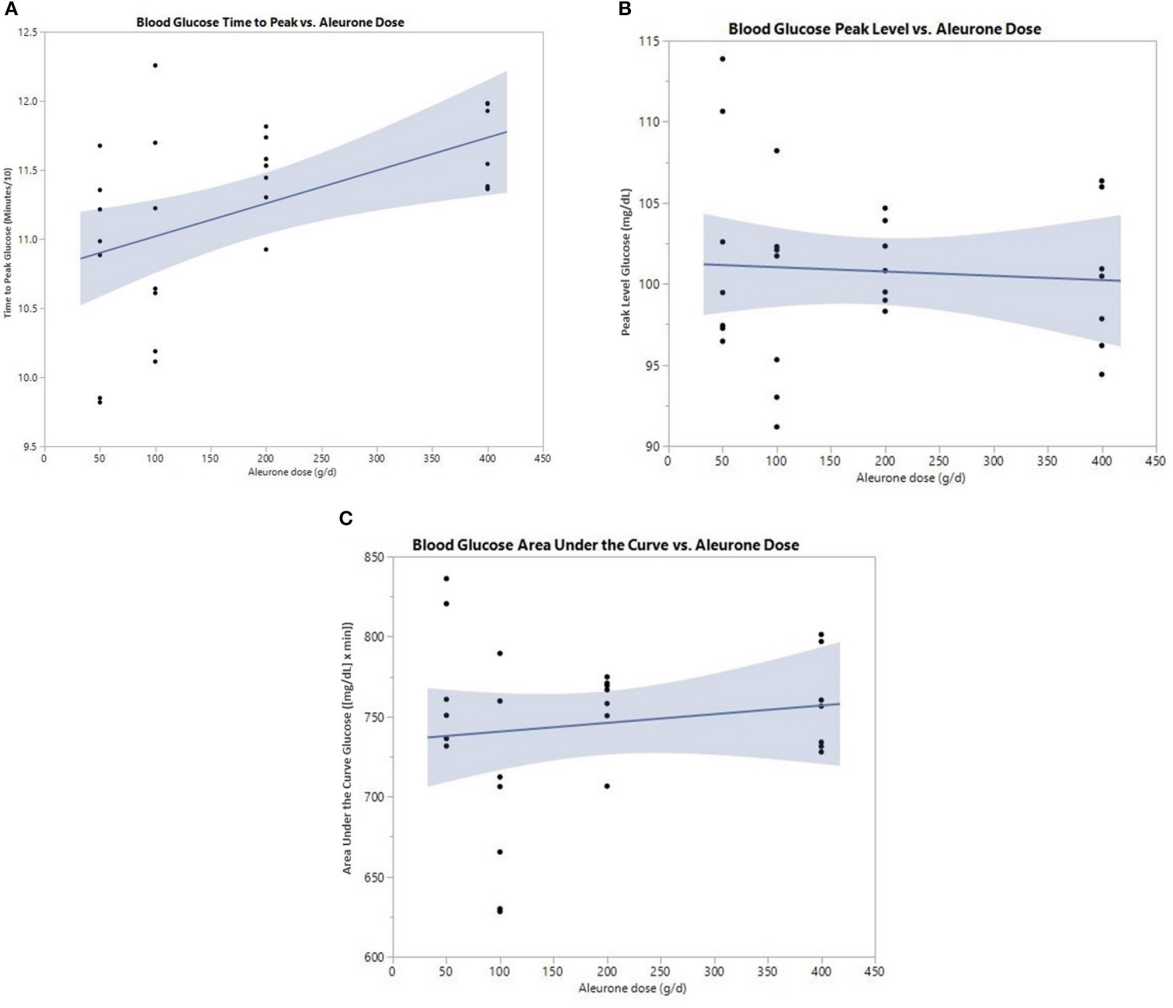
**(A)** Postprandial time to peak (min/10) of blood glucose after supplementing horses with either 0, 50, 100, 200, or 400 g of aleurone. **(B)** Postprandial peak level of blood glucose (mg/dl) after supplementing horses with either 0, 50, 100, 200, or 400 g of aleurone. **(C)** Postprandial blood glucose area under the curve (AUC) [(mg/dl) × min] after supplementing horses with either 0, 50, 100, 200, or 400 g of aleurone.

There was a significant increase in the postprandial time to peak of glucose (*p* = 0.030; Figure 1A). This effect was dose dependent and significant at a dose of 200 g of aleurone (Table 3). There were no significant changes in neither the glucose peak level nor the glucose area under the curve (AUC) for any of the used aleurone doses (Figures 1B,C).

**Table 3:** Glucose values per horse and aleurone dose.

**Horse**	**Dose (g)**	**Peak value (mg/dL)**	**Time to peak (min)**	**AUC ([mg/dL] × min)**	**Basal glucose (mg/dL)**
1	0	126	120	811.52	88
1	50	128	120	836.21	90
1	100	115	150	665.44	88
1	200	110	150	750.56	94
1	400	110	210	756.56	81
2	0	115	90	659.24	90
2	50	112	120	760.82	87
2	100	110	150	789.51	96
2	200	105	180	770.93	88
2	400	111	180	796.92	85
3	0	131	120	845.41	84
3	50	116	120	750.79	86
3	100	111	150	759.76	83
3	200	110	180	758.19	88
3	400	106	150	727.92	85
4	0	121	120	225.51	94
4	50	122	120	736.30	81
4	100	110	60	706.26	79
4	200	110	150	706.55	81
4	400	100	120	760.35	91
5	0	115	120	704.87	79
5	50	112	90	497.30	85
5	100	96	120	712.28	79
5	200	115	150	769.39	80
5	400	108	180	731.40	80
6	0	120	90	784.46	89
6	50	117	60	820.55	91
6	100	110	150	629.94	95
6	200	109	180	774.83	90
6	400	110	180	801.31	90
7	0	122	90	795.40	98
7	50	112	90	731.63	87
7	100	101	120	628.19	85
7	200	104	210	766.70	94
7	400	104	240	734.05	90

**Figure 2:**
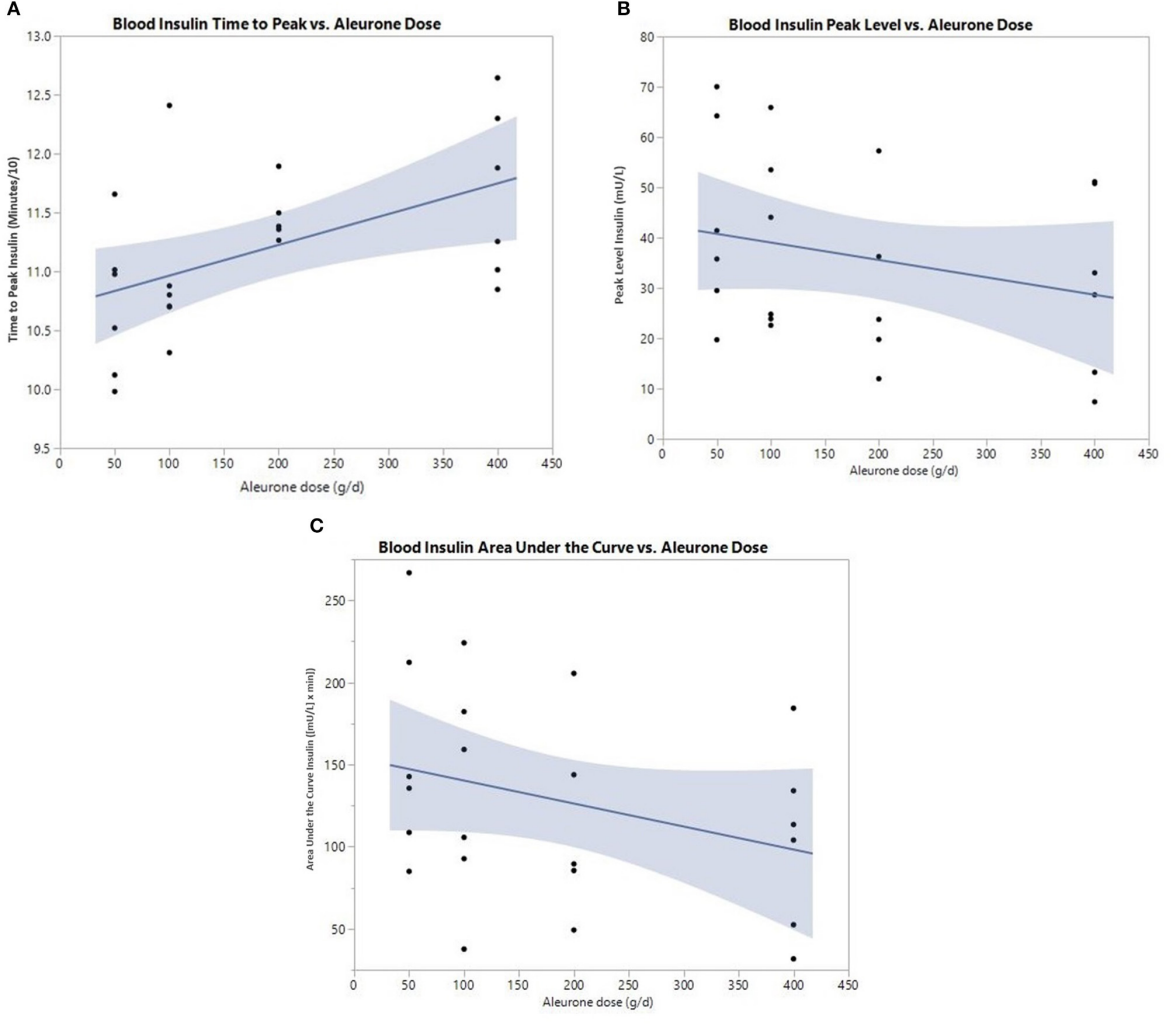
**(A)** Postprandial time to peak (min/10) of blood insulin after supplementing with either 0, 50, 100, 200, or 400 grams of aleurone. **(B)** Postprandial peak level of blood insulin (mU/L) after supplementing horses with either 0, 50, 100, 200, or 400 g of aleurone. **(C)** Postprandial blood insulin AUC [(mU/L) × min] after supplementing horses with either 0, 50, 100, 200, or 400 g of aleurone.

For insulin, all three postprandial curve parameters were significantly changed (Table 4). There was a significant delay in the time to peak of insulin (*p* = 0.025) and a significant decrease in insulin peak level (*p* = 0.049) with increasing aleurone doses, with 200 g being the lowest significant dose (Figures 2A,B). The insulin AUC was also significantly reduced (*p* = 0.019) after supplementing horses with aleurone (Figure 2C).

**Table 4:** Insulin values per horse and aleurone dose.

**Horse**	**Dose (g)**	**Peak value (mU/L)**	**Time to peak (min)**	**AUC ([mU/L] × min)**	**Basal insulin (mU/L)**
1	0	49.1	120	136.05	8.3
1	50	25.4	150	84.98	11.5
1	100	30.3	180	92.70	4.52
1	200	13.9	210	49.26	2
1	400	18.3	180	52.46	5.2
2	0				
2	50				
2	100				
2	200				
2	400				
3	0	50.7	120	167.51	3.21
3	50	32.4	150	108.66	2.26
3	100	28.5	240	37.68	
3	200	30.6	210	89.52	4.92
3	400	8.89	240	31.70	2.8
4	0	45.3	150	149.04	13.7
4	50	73.4	180	266.81	11.8
4	100	60.2	150	159.21	2
4	200	96.3	210	205.52	4.72
4	400	69.2	210	184.35	3.6
5	0	81.6	180	227.95	2
5	50	95	90	212.26	4.01
5	100	81.2	120	224.14	2.63
5	200	39.6	300	143.82	7.62
5	400	40.9	210	104.04	2.18
6	0	75	180	167.56	3.83
6	50	82.2	60	142.71	2
6	100	74.9	90	182.22	5.62
6	200	73.1	300		
6	400	61.9	240	113.50	
7	0	88.6	60	187.68	8.45
7	50	54.7	90	135.62	10.5
7	100	30.8	150	105.68	10.1
7	200	23.1	240	85.47	6.48
7	400	61.2	270	134.09	5.09

### Metagenomics

#### Alpha and Beta Diversity

Aleurone did not have any significant effects on alpha diversity or beta diversity in this study. Pairwise Kruskal–Wallis analysis using Faith's Phylogenetic diversity, Shannon's diversity, and Observed OTUs was performed but no statistical differences were detected for any alpha diversity metric. Beta diversity was assessed by using weighted and unweighted UniFrac distance as well as Bray–Curtis distance. No significant differences were found.

**Figure 3:**
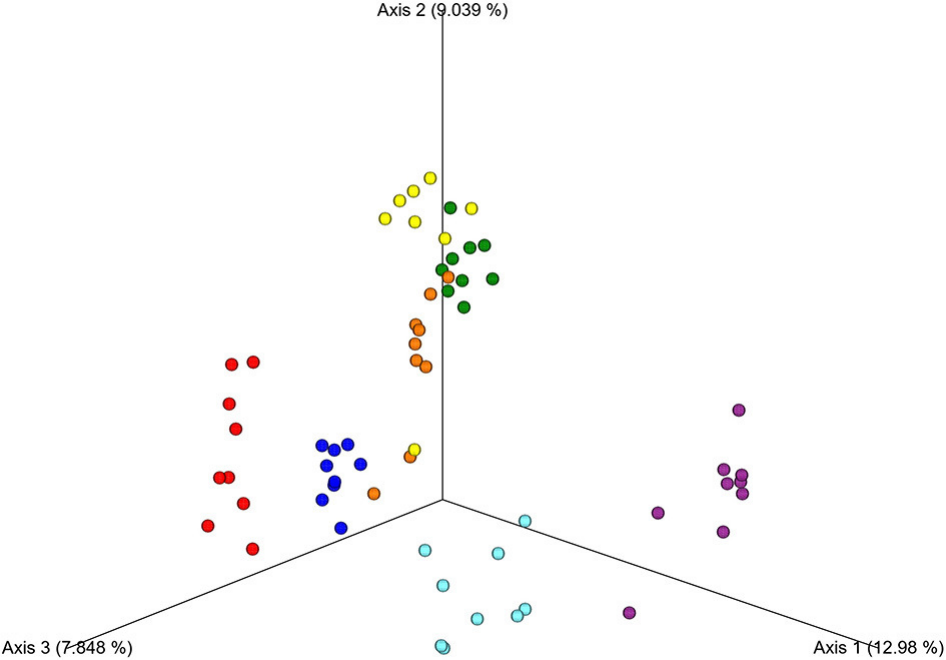
Principal Coordinates Analysis (PCoA) of the fecal samples showing the distance among the seven individual horses. The analysis was based on the weighted UniFrac metric. Different colors represent respective individual horses.

A principal coordinates analysis (PCoA) plot based on the weighted UniFrac metric (Figure 3) shows that there is large inter-individual variability of the microbiome in the studied group of horses.

#### Differentially Abundant Features

##### The Relative Abundance in Fecal Microbiome of Blanco Horses vs. Horses Supplemented With Increasing Doses of Aleurone: 50, 100, 200, and 400 g/Day

When comparing “blanco horses” with the horses after 1 week of supplementation with 50 g/day aleurone, the ANCOM analysis reveals that one unclassified species from the genus Anaerostipes decreased significantly by a factor 2.94 (W = 73 ≥ W_0.7_; FC 0.34) after aleurone supplementation. At the genus level, this change in abundance was also significantly decreased 2.98 times (W = 68 ≥ W_0.7_; FC 0.34). After a week of feeding aleurone at 100 g/day, the abundance of *Clostridium lavalense* significantly increased 2.38 times when compared to the blanco week (W = 88 ≥ W_0.7_; FC 2.38). When aleurone was supplemented at a dose of 200 g/day for 1 week, this significantly decreased the relative abundance of Shuttleworthia and Anaerostipes at both the species (W = 87 ≥ W_0.7_; FC 0.28 and W = 85 ≥ W_0.7_; FC 0.28) and genus level (W = 81 ≥ W_0.7_; FC 0.28 and W = 78 ≥ W_0.7_; FC 0.28), and increased the abundance of *C. lavalense* (W = 83 ≥ W_0.7_; FC 2.38).

##### Maximal Aleurone Dose Effect on Relative Abundance

**Figure 4:**
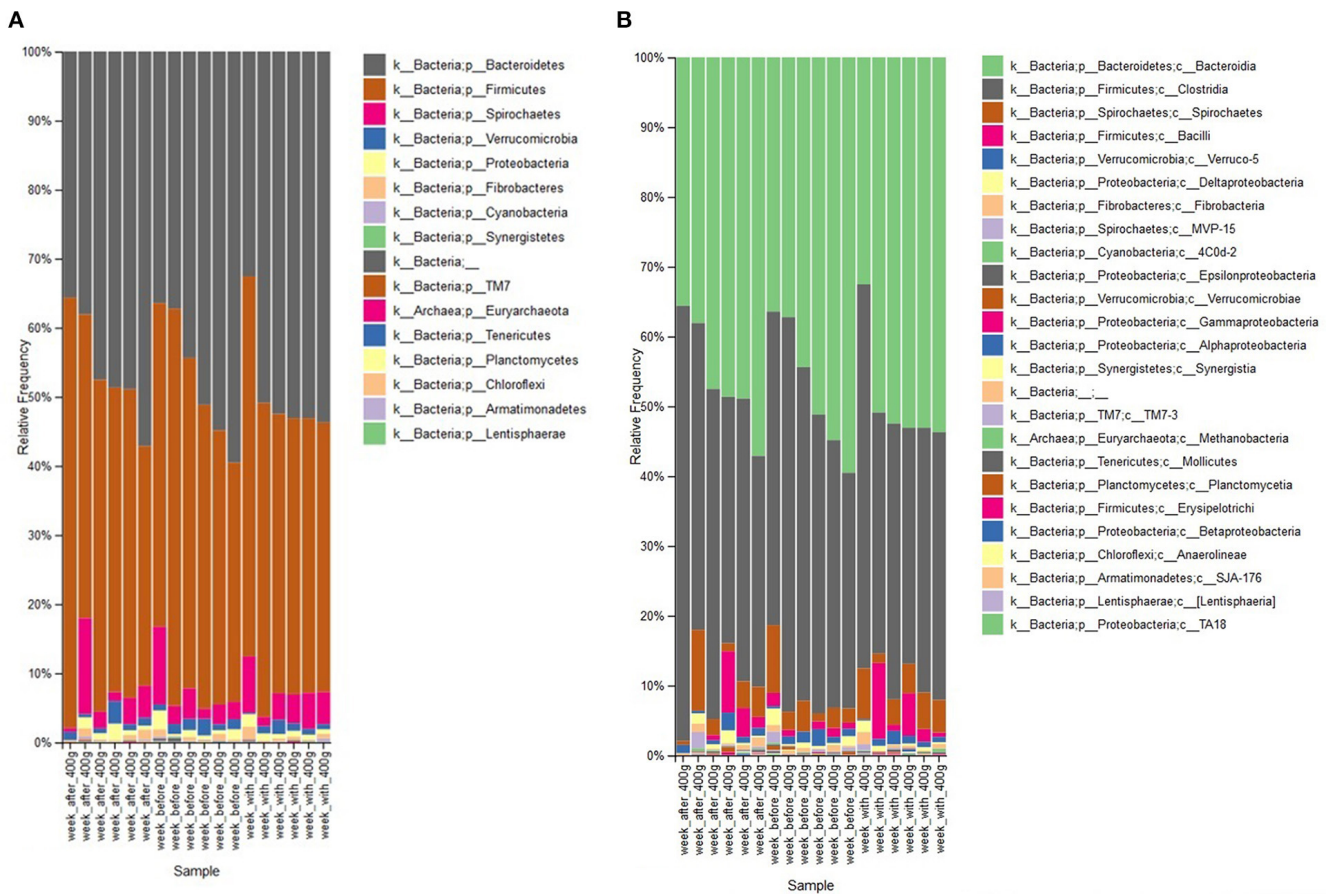
**(A)** Comparison of relative abundance of bacterial phyla present in feces of the horses that were supplemented with aleurone 400 g/day for 1 week with the relative abundance 1 week before the 400 g/day supplementation and 1 week after the 400 g/day supplementation. **(B)** Comparison of relative abundance of bacterial classes present in feces of the horses that were supplemented with aleurone 400 g/day for 1 week with the relative abundance 1 week before the 400 g/day supplementation and 1 week after the 400 g/day supplementation.

To further study the effect of the maximal dose of aleurone, the relative abundance of taxa in feces of the week before the feeding of 400 g/day was compared with their relative abundance after 1 week of 400 g/day supplementation. Feeding with 400 g/day of aleurone significantly decreased the relative abundance of an unclassified species out of the Roseburia family (W = 87 ≥ W_0.7_; FC 0.16) and an unclassified Succinovibrionaceae species (W = 72 ≥ W_0.7_; FC 0.18) was significantly decreased as well. At the genus level, Succinovibrionaceae (W = 66 ≥ W_0.7_; FC 0.18) and Shuttleworthia (W = 61 ≥ W_0.7_; FC 0.31) were significantly decreased. At the phyla level, Firmicutes, Bacteroidetes, Spirochaetes, and Verrumicrobia showed the highest changes in average relative abundance after feeding 400 g/day of aleurone (Figure 4, Table 5). Firmicutes and Verrucomicrobia decreased and Bacteroidetes and Spirochaetes increased in the relative abundance.

**Table 5:** Average relative composition at phyla level in percentage (%) 1 week before 400 g/day aleurone supplementation and after 1 week of 400 g/day of aleurone.

	**Phyla**	**Before 400 g/day (%)**	**After 7 days of 400 g/day (%)**
	Bacteroidetes	47.29	49.86
	Firmicutes	45.67	42.64
	Spirochaetes	3.81	4.62
	Verrucomicrobia	1.33	1.15
	Proteobacteria	0.88	0.88
	Fibrobacteres	0.54	0.53
	Cyanobacteria	0.14	0.12
	Synergistetes	0.06	0.04
	Unclassified	0.12	0.00
	TM7	0.02	0.05
	Euryachaeota	0.02	0.04
	Tenericutes	0.06	0.03
	Planctomycetes	0.03	0.02
	Chloroflexi	0.01	0.02
	Armatimonadetes	0.02	0.01
	Lentisphaerae	0.01	0.01

##### Long-Term Gut Microbiome Changes After Aleurone Supplementation

A significant increased abundance of an unclassified bacteria was found when comparing the 200 g/day dose with the non-supplemented post-trial week. However, the particular bacteria were unclassifiable up to the phylum level, but the effect was measurable from the species (W = 96 ≥ W_0.7_; FC 2.92) to the phylum level (W = 15 ≥ W_0.7_; FC 5.02). Other aleurone doses did not cause long term changes in abundance.

When relative abundance of ‘blanco horses’ was compared to the “posttrial” abundance, one significantly decreased species and accompanying genus were identified: an unclassified species (W = 54 ≥ W_0.7_; FC 0.11) from the genus Faecalibacterium (W = 50 ≥ W_0.7_; FC 0.11).

##### Predicted Functional Gut Microbiota Changes Induced by Aleurone Supplementation

###### Predicted functional gut microbiota of blanco horses vs. horses supplemented with different doses of aleurone

Based on the functionality predictions using PICRUSt and ANCOM2, several significantly different metabolic pathways were detected from a pairwise comparison between the samples from blanco horses compared to the horses supplemented with different doses of aleurone. Aleurone supplementation of 50 g/day significantly decreased the *lactose and galactose degradation I pathway* 49.6 times (W = 278 ≥ W_0.7_; FC 0.02). Supplementation of 100 g/day aleurone significantly changed 15 predicted pathways and supplementation of 200 g/day aleurone significantly changed 7 predicted pathways (see Table 6). When 400 g/day aleurone was supplemented, the *TCA cycle IV (2-oxoglutarate decarboxylase)* was significantly increased (W = 246 ≥ W_0.7_; FC 12).

**Table 6:** Prediction of metagenome functional content correlated with the aleurone supplementation at a level of 100 and 200 g/day using Phylogenetic Investigation of Communities by Reconstruction of Unobserved States (PICRUSt).

**Pathway description**	**100 g/day**		**200 g/day**	
	**W**	**FC**	**W**	**FC**
Superpathway of glycol metabolism and degradation	299	0.026	297	0.011
Isoprene biosynthesis II (engineered)	282	8.249	270	8.249
Coenzyme M biosynthesis I	281	8.833		
Flavin biosynthesis II (archaea)	281	8.734	264	8.734
Mevalonate pathway II (archaea)	279	10.204		
CDP-archaeol biosynthesis	277	10.072		
coenzyme B biosynthesis	276	13.058		
7-(3-amino-3-carboxypropyl)-wyosine biosynthesis	276	14.573		
Methanogenesis from H_2_ and CO_2_	275	11.155		
Phosphopantothenate biosynthesis III	274	11.596		
Archaetidylserine and archaetidylethanolamine biosynthesis	273	12.528	244	12.528
Archaetidylinositol biosynthesis	273	11.637		
Superpathway of taurine degradation	272	0.058	297	0.036
Tetrahydromethanopterin biosynthesis	272	11.575	247	11.575
Factor 420 biosynthesis	270	13.578		
Lactose and galactose degradation I			240	0.046

*W is the analysis of composition of microbiomes (ANCOM) statistic, and FC is fold change compared to no aleurone supplementation. Boxes were left empty for the non-significant W statistics (< W_0.7_) and accompanying FCs*.

###### Maximal aleurone dose effect on predicted functional Gut microbiota

Based on the functionality predictions using PICRUSt and ANCOM, two significantly different metabolic pathways were detected with a pairwise comparison between the samples collected in the week before feeding 400 g/day aleurone and at the end of 1 week of feeding 400 g/day, namely: a decrease of *superpathway of glycerol degradation to 1,3-propanediol* (W = 249 ≥ W_0.7_; FC 0.005) and a decrease of the *L-glutamate degradation VIII (to propanoate) pathway* (W = 222 ≥ W_0.7_; FC 0.114).

###### Long-term effect on predicted functional Gut microbiota after aleurone supplementation

Based on the functionality predictions using PICRUSt and ANCOM, four significantly decreased metabolic pathways were detected with a pairwise comparison between the samples collected in the “pretrial” week and the “posttrial” week. The pathway of *photorespiration* was significantly decreased (W = 302 ≥ W_0.7_; FC 0.077) in the “posttrial” week. The *lactose and galactose degradation I* pathway was significantly decreased as well (W = 292 ≥ W_0.7_; FC 0.006), and both the *methanogenesis from acetate* pathway (W = 227; FC 0.042) and the *L-glutamate degradation VIII (to propanoate)* pathway (W = 226 ≥ W_0.7_; FC 0.054) were decreased as well.

## Discussion

This is the first study focusing on the effects of aleurone supplementation in horses. A Latin square model was applied in seven horses to study the effect of different doses of aleurone on the postprandial glucose and insulin metabolism as well as the gut microbiome. Our results suggest a multimodal effect of aleurone, which is most likely to be caused by: (1) the effect of aleurone on feed texture and subsequent digestive processing; (2) the synergistic effect of the individual aleurone components on the glucose-insulin metabolism; and (3) the effect of aleurone and its components on the gut microbiome composition and metabolic output of the microbiome.

No adverse effects of aleurone supplementation were found on CBC and clinical biochemistry when feeding 400 g of aleurone/day. Also, in other animal species and humans, no adverse effects associated with aleurone supplementation have been reported (76, 77).

In the present study, aleurone supplementation induced a significant increase in the time to peak of postprandial glucose. For the insulin response, there was a significant increase in the time to peak and a significant decrease in the peak level and AUC. The delayed time to peak of postprandial glucose has not been explored in many studies, which focus on the supplementation of aleurone or one of its components (78). Several studies report an improvement of glucose homeostasis or a decrease of the peak level of glucose after an oral glucose tolerance test (OGTT) or a meal uptake supplemented with either arabinoxylan and betaine or FA (50, 79–81). The increased time to peak of glucose having an effect on the glucose peak level found in the current study is in accordance with the study of Östman et al. (78), who reported a significant delay in the time to peak of glucose after ingesting wholemeal rye bread. In addition, no change in the glucose peak level was noticed.

There are several possible explanations for the effect of aleurone on glucose-insulin metabolism. A first possible explanation for the delayed time to peak of glucose could be that insoluble and soluble dietary fibers, especially β-glucans and arabinoxylans, have their impact on the food bolus texture, which increase its viscosity. These dietary fibers are thought to form a viscous solution in the stomach, which delay gastric emptying and intestinal absorption of macronutrients. At the same time, the seeping through of digestive enzymes is delayed, which, among other things, reduces the hydrolyzation of polysaccharides (82, 83). A recent study showed that aleurone influences feeding behavior in pigs. Pigs had fewer but longer meals with a high level of dietary fiber, with an increased interval between subsequent meals, without an effect on the total daily *ad libitum* feed intake. The meal frequency significantly decreased when aleurone supplementation increased (84). Arabinoxylans are also known to blunt the gastric inhibitory peptide (GIP) release. GIP is a well-known stimulator of glucose-dependent insulin secretion (78, 85, 86). In order to investigate the effects of dietary steamed wheat bran and arabinoxylan on postprandial energy metabolism in mice, a group of researchers performed several experiments that included single feedings of a control diet vs. a steamed wheat bran diet and feeding a dietary fiber-free diet vs. different dietary concentrations of arabinoxylans. After feeding the arabinoxylan-enriched diet, a significantly lower peak level and AUC of postprandial glucose were seen when compared to the dietary fiber-free fed group (87). The results also showed lower postprandial blood GIP levels in the wheat bran fed group compared to the control diet-fed group. Wachters-Hagedoorn et al. (86) observed that the ingestion of corn pasta, a slow carbohydrate, can induce a late and prolonged GIP response when compared to the faster carbohydrates, such as glucose and corn starch in healthy men.

When comparing the results of studies on aleurone, one should keep in mind that different grain milling techniques can have their additional influence on the study results (38). In the present study, the ASP-01 type was used, which is known to have a bigger particle size when compared to ASP-02 (24). In a recent *in vitro* study, researchers tested whether there were differences in the digestion of different cell wall integrity wheat milling fractions. They found that higher integrity acted as a physical barrier to enzymes, delaying digestion (88). Different milling processes also affect the availability of the components such as betaine (89). This entails that, most probably, both the three-dimensional (3D) structure of aleurone and its particle size have an effect on how well aleurone is digested and to what degree its components are absorbed in the intestinal tract.

Besides these “physical” effects of aleurone on the digestive processing of the food bolus, several of the individual components of the aleurone fraction have been also shown to enhance postprandial insulin signaling and sensitivity as well as have an effect on glucose metabolism (33, 50, 79, 90). In a human study, Garcia et al. (79) found that after 6 weeks of supplementation with an arabinoxylan-rich diet, there is a significant decrease in the postprandial peak level of glucose, insulin, and triglycerides compared to non-supplemented subjects. Pigs fed with an arabinoxylan-enriched bread and a dark ground rye bread had lower postprandial peak levels of insulin when compared to other diets (90). Additionally, Fan et al. (50) observed that betaine supplementation to high-fructose-fed obese rats showed a reduced peak level and AUC of glucose in response to an OGTT, which suggests an improved insulin sensitivity. FA, another component of aleurone, improved the glucose homeostasis in high-fat diet-induced obese mice, and in a more recent study, these researchers showed that FA improved insulin sensitivity in a skeletal muscle of obese mice by stimulating the IRS/PI3K/Akt pathway (33). Also, the other studies looking into the physiological role of betaine report the same results (91, 92). After 12 weeks of a whole-grain rich diet intervention in humans, a reduced postprandial peak level and AUC of insulin were reported, which was inversely correlated with the levels of plasma glycine betaine (92). Likewise, Grizales et al. (91) reported a lower peak level of insulin during an OGTT in conjunction with betaine supplementation. Recently, the effect of aleurone on insulin metabolism in pigs has been described in a study where three groups of growing pigs were fed with different concentrations of aleurone or a control diet without aleurone. After 7 days, a lower peak level of postprandial insulin was reported for the highest aleurone-concentrated diet (77).

The combined expression of the reduced and delayed postprandial insulin response (time to peak, peak level, and AUC) together with the delayed glucose response suggest an increased insulin sensitivity in the aleurone supplemented horses. A similar effect has been reported in the other studies looking into the effect of aleurone or one of its components on insulin sensitivity in human and other species (91, 93–96). For instance, in pigs that were fed with aleurone-enriched bread, the mean plasma concentration of postprandial insulin was significantly lower in combination with an unchanged postprandial glucose level when compared to a diet consisting of a bread manufactured from whole wheat grain (93). In pigs that were fed with an arabinoxylan-rich diet, there was a decreased postprandial peak level of insulin and C-peptide, which indicated that less insulin was necessary to clear glucose from the bloodstream after the ingestion of a meal having high arabinoxylan content when compared to the diet poor in this dietary fiber (94). Increased insulin sensitivity has also been observed in a betaine study in an average human population where there was a strong association between the administration of choline and betaine and a lower degree of IR (95). In obese mice that were fed with a high-fat diet supplemented with betaine and subjected to an intraperitoneal insulin tolerance test, a lower level of blood glucose was observed compared to the control group (96). Quemeneur et al. (77) showed an increased insulin sensitivity after 7 days of feeding an aleurone supplemented diet to pigs, which was shown by a lower peak level of postprandial insulin reported for the highest aleurone-concentrated diet (77). In horses that were fed with a carbohydrate-rich diet having a low-level structure and composed mainly of starch and sugar, a decreased insulin sensitivity and glucose tolerance develop, which are evidenced by an increase of the AUC of insulin with an unchanged AUC of glucose during an OGTT (97). It would be interesting to involve also an IV glucose tolerance test in future equine aleurone studies. This would allow for studying aleurone effects when bypassing the GI tract. As mentioned previously, the results of the current study point toward an increased insulin sensitivity in aleurone supplemented horses, probably through a complex physiological interplay between aleurone physical properties, aleurone components, and the microbiome. More research is needed to further elucidate how this works in horses. Increased insulin sensitivity is desirable in several pathological conditions, such as equine metabolic syndrome (EMS) and laminitis (98, 99). Higher insulin sensitivity is also expected to coincide with increased performance capacity in healthy individuals since insulin is a physiological key metabolic hormone importantly involved in energy metabolism and anabolic processes (100–102).

In conjunction with the effects of aleurone supplementation on the glucose and insulin metabolism, there were also significant effects on the gut microbiome. Overall, the phyla showing the highest changes in abundance in response to aleurone supplementation were Bacteroidetes, Spirochaetes, Firmicutes, and Verrucomicrobia, of which the former two increased in abundance and the latter two decreased although these changes were not significant at the phyla level in the current study. Firmicutes and Bacteroidetes are the important phyla represented in the microbiome of a healthy horse (103), which have shown a variation in some cases and stability in others in their relative abundance in response to for instances dietary interventions such as feeding a natural high-energy forage-only diet, in which the Firmicutes and Bacteroidetes phyla varied in relative abundance (104, 105).

The results in the current study agree with a study performed by Proudman et al. (106) on healthy thoroughbred racehorses that were fed with a standard diet, where they found Firmicutes and Bacteroidetes to dominate the fecal microbiota. In the same study, they also observed an inter-individual variation in response to dietary change. The results in the present study also agree with the results of a study in which healthy horses were fed with fiber-based diets, and at the end the researchers found the most abundant phyla to be Bacteroidetes followed by Firmicutes, with smaller quantities of Spirochaetes, Fibrobacteres, Proteobacteria, and Actinobacteria (107).

The other way around, a decrease in Bacteroidetes is reported to be associated with unfavorable conditions. When comparing prepartum and postpartum mares' fecal microbiota, a decrease in the relative abundance of the phylum Bacteroidetes (5.2 vs. 2.1%) was significantly associated with the development of colic caused by large colon volvulus, gas colic, or other large colon displacements. In the current study that involved healthy non-pregnant horses, Bacteroidetes levels were much higher at the start of the study (49.9%), and they further increased during aleurone supplementation (52.7%). Furthermore, postpartum mares were also more prone to develop colic when the relative abundance of the phylum Firmicutes was lower than 50%. Interestingly, the mean pretrial and posttrial Firmicutes abundance was much lower in our study (45.7% before 400 g/day of aleurone supplementation and 42.6% after 400 g/day of aleurone supplementation) when compared to, respectively, 68% pre-partum and 58% postpartum in the study of Weese et al. (108). It can be expected that “basal” fecal microbiome composition of a certain herd is determined by many factors, such as differences in dietary management (concentrate feed, roughage, and pasture turnout), stable management, applied training intensities, and even hormonal influences (108–110).

A recent study described a difference in the community structure of the microbiota between healthy horses and the horses suffering from EMS. EMS horses showed a significantly lower microbiota diversity. However, this study could not identify significant differences caused by EMS at the phyla level or lower taxonomic order (111). Interestingly, obese individuals showed an increase in the relative abundance of the phylum Firmicutes and a decrease of the phylum Bacteroidetes as well (112, 113).

Dougal et al. (107) performed a weight-loss study involving obese horses and ponies and showed a significant decrease in the relative abundance of the phylum Firmicutes and an increase of the phylum Bacteroidetes in association with weight loss. Assuming that these horses developed an increased insulin sensitivity during the study while losing weight, this microbiome change would be in accordance with the results of the current study. However, the obese horses were only sampled at week 10 and 16 of the dietary intervention and not at the beginning of the weight-loss program. In addition, no follow-up of neither glucose metabolism nor insulin metabolism was performed (107). Morrison et al. (114) studied the effect of weight loss induced by 7 weeks of dietary restriction on ponies' fecal microbiome and found a significant decrease of several bacterial genera including the genus Roseburia. Aleurone decreased this genus of the Lachnospiraceae family in the current study as well (114). Shuttleworthia and Anaerostipes, both genera from the Lachnospiracaea family, and the carbohydrate-fermenting family of Succinovibrionaeciae were also decreased by aleurone supplementation. High abundance of Anaerostipes is associated with type 2 diabetes mellitus in humans and in high insulin resistant humans the abundance of Succinovibrionaeciae was positively correlated to the degree of inflammation of the visceral adipose tissue (115, 116).

Apart from associating changes in body weight with unfavorable changes in the microbiome, researchers have also found a correlation between the insulin regulation and certain microbiome compositions in ponies with insulin dysregulation. An introduction of pasture turnout induced a significant change in the differential abundance of the fecal microbiota of these ponies. A multivariate regression analysis showed that the insulin sensitivity status of each pony accounted for a more variation in the fecal microbiome than the variation typically observed between horses. Adding pasture turnout to the diet accounted for 3% of the fecal microbiome variation. However, strikingly, the insulin status of these ponies accounted for 15.1% of the microbial variation. The Ruminococcaceae family, on the other hand, was decreased as well in the fecal microbiome of the insulin dysregulated ponies, which is in accordance with our study in which the genus of the butyrate producer Faecalibacter from the family of Ruminococcaceae decreased as well. The relative abundance of the Lachnospiraceae family on the other hand, was significantly decreased in insulin dysregulated ponies, a change that was not found in the current study (110).

In a recent equine 2-year weight gain study looking into the effect of obesity on the equine fecal microbiome, a significant increase of the relative abundance of the phylum Firmicutes was shown to be associated with increased body weight, body condition score, and cresty neck score. In addition, the phyla Bacteroidetes and Spirochaetes both decreased in relative abundance in horses and ponies with an increase in obesity, although these differences were not significant (117). Assuming that these horses developed insulin dysregulation while gaining weight, this fecal microbiome change is in accordance with our data as well: with increasing insulin sensitivity, the relative abundance of the phyla Bacteroidetes and Spirochaetes is increased, and the relative abundance of the phylum Firmicutes is decreased.

Finally, it is also important to realize that the changed metabolome of the changed gut microbiome can have its effects on the findings in the current study. Indeed, it can be expected that changes in the composition of the gut microbiome lead to the changes in the metabolic fingerprint of the microbiome (106). Some of these metabolites have already been identified as having beneficial health effects. For example, it has been established that, as a part of the dietary fiber fraction, aleurone is partly digested by the gut microbiota, which leads to the production of short-chain fatty acids (SCFAs) [i.e., acetate, butyrate, and propionate; (118–120)]. These SCFAs could modulate health benefits both locally at the level of the gastrointestinal tract (121), and systemically, for example, through the amelioration of insulin sensitivity (122–124). Other by-products in the fermentation of fibers using the gut microbiota are lactate, valerate, aromatic amino acids (AAAs), and branched-chain amino acids (BCAAs) (125–127). It would be interesting to look into shifts in the metabolome of the gut microbiome in response to aleurone supplementation in future studies.

The PICRUSt analysis in this study showed several significant predicted effects of aleurone on the functional fecal metabolome energy pathways. The *L-glutamate degradation VIII pathway* was significantly downregulated after 400 g/day of aleurone supplementation and in the “posttrial” fecal samples as well. This pathway ferments glutamate into propionate and is expected to be mainly used by bacteria from the phylum Firmicutes. Aleurone was predicted to downregulate the propionate production by the gut bacteria in this study, which could lead to decreased levels of endocrine hormone glucagon-like-peptide 1 (GLP-1). GLP-1 increases insulin serum levels and it is possible that an aleurone-induced decrease in propionate plays a role in the observed effects on the glucose-insulin metabolism as seen in the current study (128). A different amino acid degradation pathway that was downregulated by aleurone is the *Super pathway of taurine degradation*, which would provide the horse with more taurine to absorb from its gut. Taurine is a natural occurring amino acid part of bile acid conjugation, osmoregulation, membrane stabilization, and regulation of intracellular calcium homeostasis and is known to protect equine lymphocytes against oxidative stress (129). Moreover, taurine has an effect on the beta-cell insulin secretion in hamster cell-line and rat cell-line by changing beta-cell current (130). Oral supplementation of taurine improved insulin sensitivity and controlled hyperglycemia and hyperinsulinemia in fructose-fed insulin resistant rats by altering the insulin signaling-enzymes protein tyrosine kinase and protein tyrosine phosphatase in the liver; therefore modifying post-receptor events of insulin action. Kim et al. (131) have shown that taurine-ameliorated hyperglycemia and dyslipidemia in insulin resistant rats by decreasing IR and leptin levels (132). It is possible that the effects of aleurone on the gut microbial metabolome, including taurine production, are part of the explanation of how aleurone modifies postprandial glucose-insulin levels.

Three consecutive pathways expressed by the domain of Archaea, all contributing to phospholipid membrane synthesis, were significantly upregulated by aleurone. This included the pathways of *CDP-archaeol biosynthesis, Archaetidylserine and archaetidylethanolamine biosynthesis*, and *Archaetidylinositol biosynthesis*. The function of the entire domain of Archaea in the equine gut microbiome and metabolome is not well-studied (133). The biologic production of methane, an anaerobic respiration process called methanogenesis, is carried out by this domain and feeding of aleurone induced a shift from *Methanogenesis from acetate* to *Methanogenesis from H*_2_ *and CO*_2_ by these methanogens. This can possibly save more of the SFCA acetate for either the equine energy metabolism or as a nutrition for the gut microbiome and simultaneously decreasing luminal H_2_ and CO_2_. Several necessary cofactors of the upregulated pathway of *Methanogenesis from H*_2_ *and CO*_2_ were also predicted to be expressed at a higher level after aleurone supplementation: including *Cofactor 420 biosynthesis I, Coenzyme M biosynthesis I*, and *Coenzyme B biosynthesis*. Aleurone possibly induces the growth of the Archaea domain by inducing the membrane formation and pushes the methanogenesis into using H_2_ and CO_2_ instead of the SFCA acetate.

In conclusion, the results of this study show that aleurone blunts the postprandial glucose and insulin response and induces significant shifts in the gut microbiome. Our results suggest a multimodal effect of aleurone, which is most likely caused by the effect of aleurone on the feed texture and subsequent digestive processing, a synergistic effect of the individual aleurone components on the glucose-insulin metabolism, and the effect of aleurone and its components on the gut microbiome composition and metabolic output of the microbiome. More research is needed to further unravel the background of these findings.

## Data Availability Statement

The datasets presented in this study can be found in online repositories. The name of the repository and accession number can be found at: https://www.ebi.ac.uk/ena. The study ID is PRJEB42805 (ERP126716).

## Ethics Statement

The animal study was reviewed and approved by the Animal Ethics Committee of the Ghent University EC 2014.14. Written informed consent was obtained from the owners for the participation of their animals in this study.

## Author Contributions

BB and CD contributed to the conception, design, data acquisition, data analysis and interpretation, and drafting of the manuscript. CMo contributed to data analysis, data interpretation, and drafting of the manuscript. JO contributed to the conception, data analysis and interpretation, and reviewing of the manuscript. YG, DD, FV, and GH contributed to data analysis and reviewing of the manuscript. LD and CMe contributed to data interpretation and reviewing of the manuscript. All authors contributed to the article and approved the submitted version.

## Conflict of Interest

JO and GH are employed by Cargill R&D Centre Europe. Cargill has exclusivity over distribution of aleurone for feed market in Europe produced according to patents held by Buhler Company. The remaining authors declare that the research was conducted in the absence of any commercial or financial relationships that could be construed as a potential conflict of interest.
