# Supplementary material for: Effects of Aleurone Supplementation on Glucose-Insulin Metabolism and Gut Microbiome in Untrained Healthy Horses
Source: Front Vet Sci. 2021 Apr 12;8:642809. doi: 10.3389/fvets.2021.642809 (PMC8072273; doi:10.3389/fvets.2021.642809)
Supplement: Supplementary file 1 [file Table_1.docx]

| **Supplementary Table 1** | |
| --- | --- |
| **Blanco feed** | |
| **Raw materials** | **Amount (%)** |
| Wheat short flour | 29,982 |
| Barley | 21,000 |
| Soybeans 30% RC | 11,000 |
| Alfalfa for horses | 10,500 |
| Fine corn flakes RV 11 | 8,000 |
| Wheat nt. Grain (flour) | 6,525 |
| Molasses P67 | 4,000 |
| Palm oil | 2,475 |
| Fine chalk | 2,275 |
| Maize | 2,000 |
| Salt | 0,900 |
| Dry matter MD | 0,500 |
| Premix horse 0,25% | 0,400 |
| Sodium bicarbonate | 0,288 |
| MgO 90% | 0,125 |
| Vit E 50% | 0,030 |
| Total | 100,000 |
| **Nutrients** | **Amount in product** |
| Dry matter | 875,982 |
| Moisture | 124,018 |
| Crude ash | 77724,000 |
| Crude protein | 111,260 |
| Crude fat | 55,068 |
| Crude fiber | 105,091 |
| Other carbohydrates | 524,069 |
| Starch | 282,780 |
| Sugars | 59,396 |
| Sugars and starch | 342,123 |
| Horse Feed Unit | 825,987 |
| EWpa^[[1]](#footnote-1)^ | 0,701 |
| FOS g/kg | 497,487 |
| Digestible crude protein | 85,562 |
| Lysine | 4,638 |
| Methionine | 1,789 |
| NSP g/kg | 312,479 |
| Na+ K-Cl meq/kg | 270,116 |
| Calcium | 12,449 |
| Potassium | 9,798 |
| Sodium | 4,505 |
| Chlorine | 6,408 |
| Magnesium | 2,627 |
| Total phosphorus | 4,315 |
| Selenium (mg/kg) | 0,544 |
| Sodium selenite (E8)/selenium | 0,544 |
| Iron (mg/kg) | 340,291 |
| Iron (II) sulfate monohydrate (E1)/iron | 200,000 |
| Manganese (mg/kg) | 119,176 |
| Manganese (II) oxide (E5)/manganese | 80,000 |
| Zinc (mg/kg) | 158,944 |
| Zinc oxide (E6)/zinc | 120,000 |
| Copper (mg/kg) | 39,504 |
| Copper (II) sulfate pentahydrate (E4)/copper | 34,000 |
| Iodine (mg/kg) | 1,059 |
| Anhydrous calcium iodate (E2)/iodine | 1,000 |
| Cobalt (mg/kg) | 0,891 |
| Cobalt (II) carbinate hydroxide (2:3) monohydrate | 0,000 |
| C18:2 g/kg | 14,567 |
| C18:3 g/kg | 1,327 |
| Vitamin A (E672) | 25000,000 |
| Vitamin D3 (E671) | 3000,000 |
| Vitamin E (All-rac-alpha-tocopheryl acetate) | 150,000 |
| Vitamin K3 | 4,000 |
| Vitamin B1 | 20,000 |
| Vitamin B2 | 25,000 |
| Calcium-D-Pantothenate | 30,000 |
| Vitamin B6 | 10,000 |
| Vitamin B9 | 4,000 |
| Vitamin B12 | 64,000 |
| Vitamin PP (Niacin) | 60,000 |
| Vitamin C | 0,000 |
| Biotin | 304,000 |

| **Aleurone concentrate** | |
| --- | --- |
| **Raw materials** | **Amount (%)** |
| Wheat aleurone | 20,000 |
| Barley | 20,045 |
| Soybeans 30% RC | 12,375 |
| Alfalfa for horses | 10,500 |
| Fine corn flakes RV 11 | 8,000 |
| Wheat short flour | 7,100 |
| Wheat nt. Grain (flour) | 4,775 |
| Molasses P67 | 4,000 |
| Palm oil | 2,425 |
| Fine chalk | 2,300 |
| Maize | 6,325 |
| Salt | 0,900 |
| Dry matter MD | 0,500 |
| Premix horse 0,25% | 0,400 |
| Sodium bicarbonate | 0,200 |
| MgO 90% | 0,125 |
| Vit E 50% | 0,030 |
| Total | 100,000 |
| **Nutrients** | **Amount in product** |
| Dry matter | 876,333 |
| Moisture | 123,667 |
| Crude ash | 76,651 |
| Crude protein | 113,603 |
| Crude fat | 54,997 |
| Crude fiber | 110,350 |
| Other carbohydrates | 525,339 |
| Starch | 260,134 |
| Sugars | 55,735 |
| Sugars and starch | 315,834 |
| Horse Feed Unit | 830,920 |
| EWpa^1^ | 0,708 |
| FOS g/kg | 493,734 |
| Digestible crude protein | 83,382 |
| Lysine | 4,567 |
| Methionine | 1,770 |
| NSP g/kg | 312,386 |
| Na+ K-Cl meq/kg | 255,069 |
| Calcium | 12,499 |
| Potassium | 10,423 |
| Sodium | 4,581 |
| Chlorine | 6,425 |
| Magnesium | 3,325 |
| Total phosphorus | 5,312 |
| Selenium (mg/kg) | 0,544 |
| Sodium selenite (E8)/selenium | 0,544 |
| Iron (mg/kg) | 340,559 |
| Iron (II) sulfate monohydrate (E1)/iron | 200,000 |
| Manganese (mg/kg) | 116,138 |
| Manganese (II) oxide (E5)/manganese | 80,000 |
| Zinc (mg/kg) | 157,778 |
| Zinc oxide (E6)/zinc | 120,000 |
| Copper (mg/kg) | 39,271 |
| Copper (II) sulfate pentahydrate (E4)/copper | 34,000 |
| Iodine (mg/kg) | 1,064 |
| Anhydrous calcium iodate (E2)/iodine | 1,000 |
| Cobalt (mg/kg) | 0,893 |
| Cobalt (II) carbinate hydroxide (2:3) monohydrate | 0,000 |
| C18:2 g/kg | 14,940 |
| C18:3 g/kg | 1,313 |
| Vitamin A (E672) | 25000,000 |
| Vitamin D3 (E671) | 3000,000 |
| Vitamin E (All-rac-alpha-tocopheryl acetate) | 150,000 |
| Vitamin K3 | 4,000 |
| Vitamin B1 | 20,000 |
| Vitamin B2 | 25,000 |
| Calcium-D-Pantothenate | 30,000 |
| Vitamin B6 | 10,000 |
| Vitamin B9 | 4,000 |
| Vitamin B12 | 64,000 |
| Vitamin PP (Niacin) | 60,000 |
| Vitamin C | 0,000 |
| Biotin | 304,000 |

1. The EWpa system, the actualised Dutch net energy system for horses - PDF Free Download. Available at: https://docplayer.net/21538482-The-ewpa-system-the-actualised-dutch-net-energy-system-for-horses.html [Accessed March 31, 2020]

2. Kienzle E, Zeyner A. The development of a metabolizable energy system for horses. *J Anim Physiol Anim Nutr (Berl)* (2010) **94**: doi:10.1111/j.1439-0396.2010.01015.x

3. Ebert, M., and Moore-Colyer MJS. The energy requirements of performance horses in training.

1. EWpa means energiewaarde paarden. This measurement is the one set by the Central Bureau, Livestock Feeding, Netherlands (CVB) in 2016 as a net energy system for horses and it uses a standard value of oats as energy unit (1). EWpa is calculated to metabolizable energy (ME, German measurement) by using the CVB ME value of 1kg of oats, 11.4 MJ, and converting metabolic body weight to body weight on the basis of a 500kg horse (2,3). [↑](#footnote-ref-1)
