# Supplementary material for: Effects of Aleurone Supplementation on Glucose-Insulin Metabolism and Gut Microbiome in Untrained Healthy Horses
Source: Front Vet Sci. 2021 Apr 12;8:642809. doi: 10.3389/fvets.2021.642809 (PMC8072273; doi:10.3389/fvets.2021.642809)
Supplement: Supplementary file 2 [file Data_Sheet_1.docx]

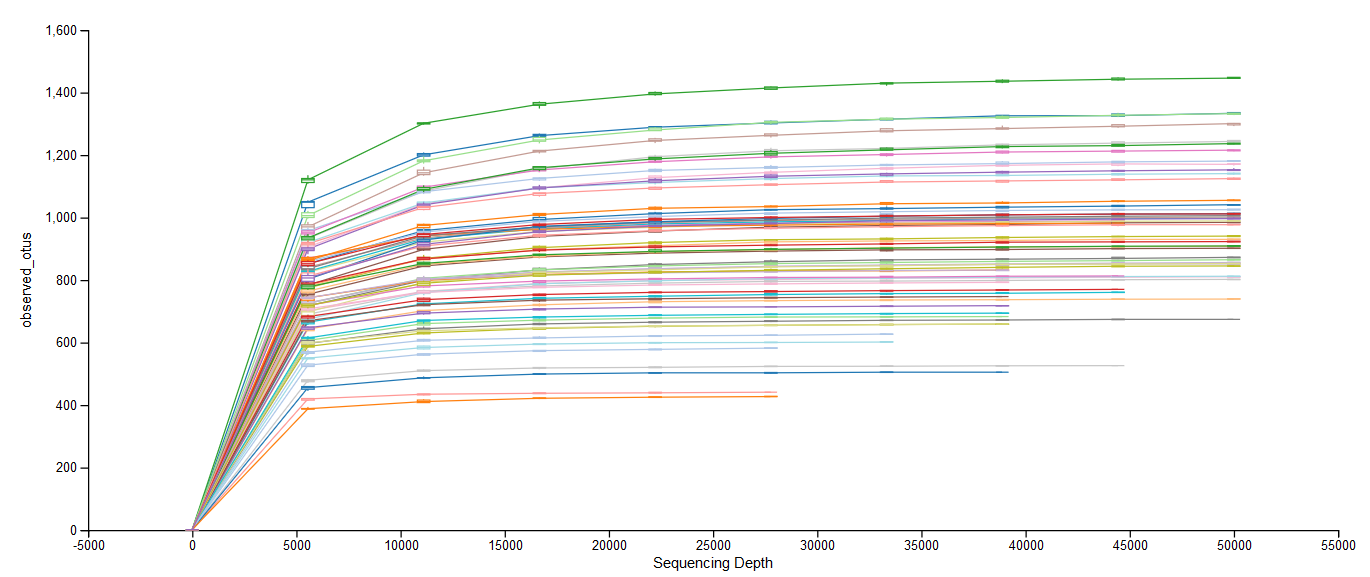
SUPPLEMENTARY FIGURE 1 Alpha rarefaction plot showing the number of unique OTUs at an increasing number of randomly sampled sequences for each fecal sample.
